# Comprehensive meta-QTL analysis for dissecting the genetic architecture of stripe rust resistance in bread wheat

**DOI:** 10.1186/s12864-023-09336-y

**Published:** 2023-05-12

**Authors:** Sandeep Kumar, Dinesh Kumar Saini, Farkhandah Jan, Sofora Jan, Mohd Tahir, Ivica Djalovic, Dragana Latkovic, Mohd Anwar Khan, Sundeep Kumar, V. K. Vikas, Upendra Kumar, Sundip Kumar, Narendra Singh Dhaka, Om Parkash Dhankher, Sachin Rustgi, Reyazul Rouf Mir

**Affiliations:** 1 Division of Genetics and Plant Breeding, Faculty of Agriculture, SKUAST-Kashmir, Wadura, 193201 India; 2 Department of Plant Breeding and Genetics, Punjab Agricultural University, Ludhiana, 141004 Punjab India; 3 Institute of Field and Vegetable Crops, National Institute of the Republic of Serbia, Maxim Gorki 30, Novi Sad, Serbia; 4 Department of Field and Vegetable Crops, Faculty of Agriculture, University of Novi Sad, Trg Dositeja Obradovića 8, 21000 Novi Sad, Serbia; 5 Indian Council of Agricultural Research-National Bureau of Plant Genetic Resources, New Delhi, India; 6 ICAR-IARI, Regional Station, Wellington, 643 231 The Nilgiris India; 7 Department of Molecular Biology & Biotechnology., CCS Haryana Agriculture University, Hisar, India; 8 Department of Molecular Biology and Genetic Engineering, Molecular Cytogenetics Laboratory, College of Basic Science and Humanities, G. B. Pant University of Agriculture and Technology, Pantnagar-263145, U.S. Nagar, Uttarakhand India; 9 Department of Genetics and Plant Breeding, College of Agriculture, G. B. Pant, University of Agriculture & Technology, Pantnagar-263145, U. S. Nagar, Uttarakhand India; 10 School of Agriculture, University of Massachusetts Amherst, Stockbridge Amherst, MA 01003 USA; 11 Department of Plant and Environmental Sciences, Clemson University, 2200 Pocket Road, Florence, SC 29506 USA

**Keywords:** Stripe rust, Meta-analysis, Consensus map, MQTL, Candidate genes

## Abstract

**Background:**

Yellow or stripe rust, caused by the fungus *Puccinia striiformis* f. sp. *tritici* (*Pst*) is an important disease of wheat that threatens wheat production. Since developing resistant cultivars offers a viable solution for disease management, it is essential to understand the genetic basis of stripe rust resistance. In recent years, meta-QTL analysis of identified QTLs has gained popularity as a way to dissect the genetic architecture underpinning quantitative traits, including disease resistance.

**Results:**

Systematic meta-QTL analysis involving 505 QTLs from 101 linkage-based interval mapping studies was conducted for stripe rust resistance in wheat. For this purpose, publicly available high-quality genetic maps were used to create a consensus linkage map involving 138,574 markers. This map was used to project the QTLs and conduct meta-QTL analysis. A total of 67 important meta-QTLs (MQTLs) were identified which were refined to 29 high-confidence MQTLs. The confidence interval (CI) of MQTLs ranged from 0 to 11.68 cM with a mean of 1.97 cM. The mean physical CI of MQTLs was 24.01 Mb, ranging from 0.0749 to 216.23 Mb per MQTL. As many as 44 MQTLs colocalized with marker–trait associations or SNP peaks associated with stripe rust resistance in wheat. Some MQTLs also included the following major genes- *Yr5*, *Yr7*, *Yr16*, *Yr26*, *Yr30*, *Yr43*, *Yr44*, *Yr64*, *YrCH52*, and *YrH52*. Candidate gene mining in high-confidence MQTLs identified 1,562 gene models. Examining these gene models for differential expressions yielded 123 differentially expressed genes, including the 59 most promising CGs. We also studied how these genes were expressed in wheat tissues at different phases of development.

**Conclusion:**

The most promising MQTLs identified in this study may facilitate marker-assisted breeding for stripe rust resistance in wheat. Information on markers flanking the MQTLs can be utilized in genomic selection models to increase the prediction accuracy for stripe rust resistance. The candidate genes identified can also be utilized for enhancing the wheat resistance against stripe rust after in vivo confirmation/validation using one or more of the following methods: gene cloning, reverse genetic methods, and omics approaches.

**Supplementary Information:**

The online version contains supplementary material available at 10.1186/s12864-023-09336-y.

## Background

Stripe (or yellow) rust, caused by *Puccinia striiformis* f. sp. *tritici* (*Pst*), is a severe biotic stress that restricts wheat production and productivity on a global scale. The yellow rust epidemics are stirring in almost every wheat growing region in the world and may result in major (up to 70%) yield losses, mainly owing to the reduced grain filling duration under severe epidemic conditions [1]. When the infection occurs at the seedling stage and environmental conditions are favorable for the pathogen to persist till maturity, yield losses may even approach 100% [2, 3]. Stripe rust proliferates because the pathogen responsible is an excellent air traveler and, as a result of its reproducing ability, it can spread over large distances in favorable climatic conditions [4, 5]. An earlier study estimated that 88% of the world’s wheat production is now prone to stripe rust infection, resulting in global losses of at least 5 million tonnes annually [6].

The development of rust-resistant wheat varieties is the most cost-effective strategy being adopted throughout the world for managing most of the plant diseases including wheat rusts [7, 8]. Therefore, it is important to map the target genes/QTLs for stripe rust in wheat followed by their introgressions into wheat varieties for enhancing their stripe rust resistance [9, 10]. The resistance to stripe rust can be divided into two categories based on the growth stage at which it appears: seeding (or all-stage) resistance and adult-plant resistance (APR, including high-temperature APR) [5, 11].

To date, eighty-three *Yr* genes for stripe rust resistance have been formally designated in wheat, and they are positioned across all 21 wheat chromosomes [12]. More than 15 of these *Yr* genes have been derived from the wild species [13]. In addition, several important *Yr* genes, including *Yr5*/*Yrsp*, *Yr7*, *Yr10*, *Yr15*, *Yr18*, *Yr36*, *Yr46 and YrU1* have been successfully cloned using a variety of approaches and are now being used in wheat breeding programs world-wide [14–17]. Major epidemics have occurred after the introduction and expansion of new virulent disease races because some of these genes have been extensively targeted in wheat breeding [18, 19]. The lessons from these occurrences were the broad use of a single major resistance gene ultimately fails, with negative consequences inversely correlated with the distribution of wheat cultivars containing that gene. Sources with improved durability were typically deployed with the intention of preventing the overuse of individual resistance genes and avoiding the deployment of combinations of effective major genes for disease resistance. Further, such resistant genotypes exhibit hypersensitivity or programmed cell death [19]. In contrast, quantitative resistance, which causes the reduction, but not absence of disease, is based on minor genes encoding various resistance responses that are not restricted to specific pathogen races.

QTL mapping is a powerful technique for the genetic dissection of complex traits, including quantitative disease resistance, in crop plants [20, 21]. Since the publication of the first study on QTL mapping for stripe rust resistance in wheat in 2000 [22], a large number of QTL mapping studies for stripe rust resistance in wheat have been published (http://wheatqtldb.net/, [23]). However, the deployment of these resistance QTLs in wheat breeding programs has been minimal for several reasons, a few of which are as follows: inconsistencies between the QTLs reported in different studies, use of different types of markers, population types and sizes, and phenotyping environments. Furthermore, as a result of the complex genome of hexaploid wheat, the presence of highly repetitive sequences in the genome and the lack of high-resolution linkage maps, less progress has been made in fine mapping and cloning of gene/QTLs. In recent years, a revolution in the QTL analysis of complex traits has occurred via the introduction of different high-throughput sequencing and genotyping technologies, thus also facilitating the effective use of genome-wide association studies (GWAS) for trait dissection [24, 25]. GWAS is a powerful and simple way to fine map QTLs in a large population by deriving multi-allelic variations to help in identifying the most advantageous alleles of a target trait in a single analysis, and it is more powerful and simple to fine map QTLs due to higher resolution resulting from high genetic diversity [26–28].

An emerging and a relatively new method known as meta-analysis of QTLs or MQTL analysis has been able to produce more reliable QTLs for MAS and prediction of candidate genes (CGs) associated with the trait in question. The reliability, feasibility and utility of MQTL analysis has already been well established in different crop plants [29–36]. The software program BioMercator [37], generally used for MQTL analysis, allows the analysis of large number of QTLs from diverse sources and helps in the projection of QTLs to a consensus or reference map [38]. As a result, meta-analysis is able to identify the consensus QTLs associated with the targeted traits in multiple environments and diverse genetic backgrounds [39]. For instance, meta-analysis has already been conducted in wheat for a number of different traits [40–44] including resistance to different diseases such as leaf rust [45, 46], stem rust [47], tanspot [48], Fusarium head blight [49–52] and powdery mildew [53]. Despite the fact that there are over 500 QTLs associated with stripe rust resistance, a recent study published in 2021 conducted a meta-analysis using only 184 QTLs and identified 61 MQTLs [54]. Considering this, the current study involving meta-analysis (based on almost all QTL mapping studies available) was planned to provide more reliable MQTLs and candidate genes for stripe rust resistance.

Overall, the present study was conducted to investigate the genetic architecture that drives resistance to stripe rust by finding intriguing candidate genes from the recently decoded wheat genome, integrating this information with transcriptome data and GWAS, and investigating the function of the identified CGs in different wheat tissues. The outcome of this study will prove useful for wheat breeding community and provide tools for improving stripe rust resistance in wheat cultivars.

## Results

### QTLs and their distribution on wheat chromosomes

**Fig. 1:**
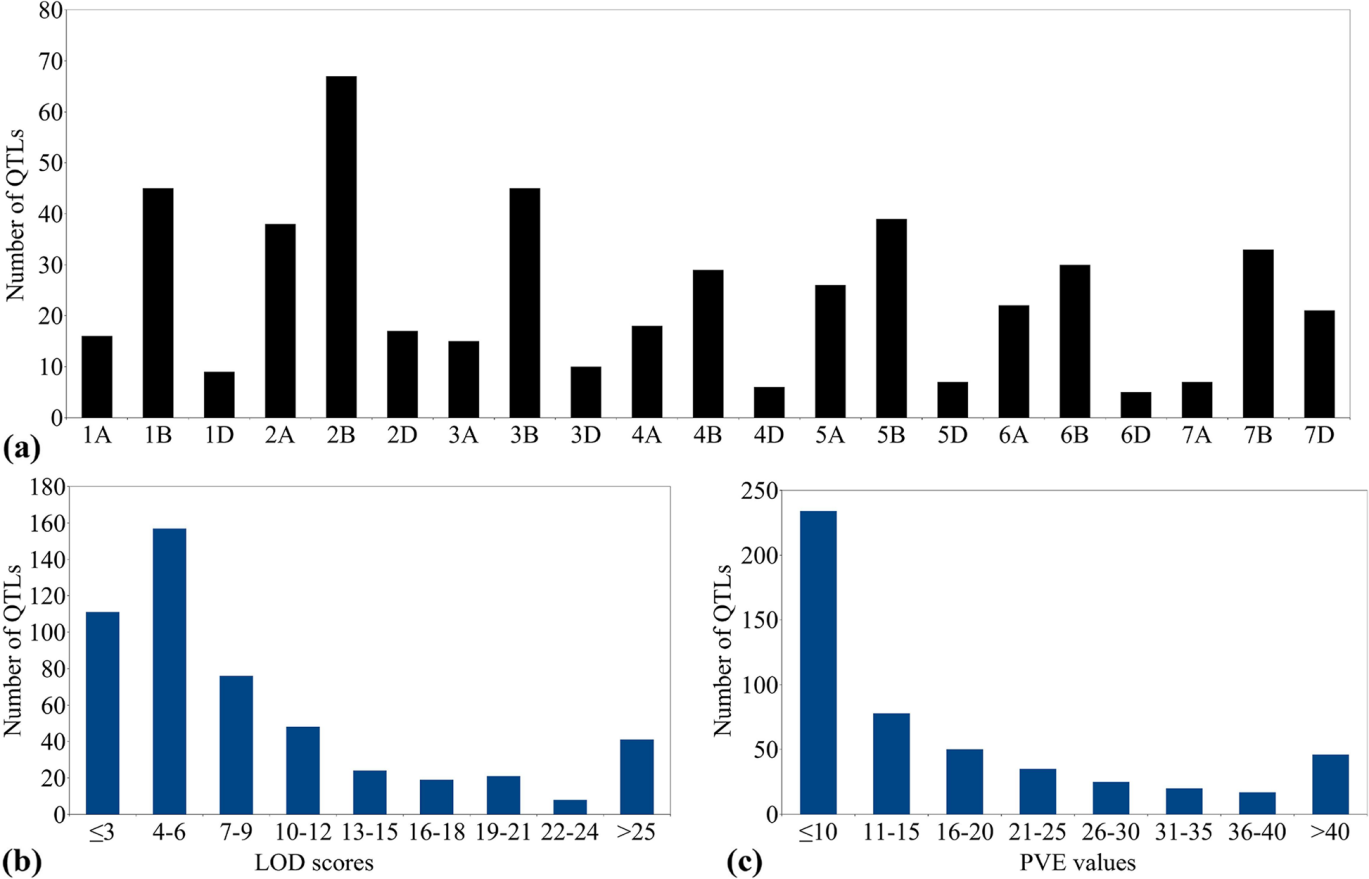
Basic characteristics of QTLs associated with yellow rust resistance (**a**) chromosome-wise distribution of QTLs, (**b)** LOD scores of QTLs, (**c)** PVE values of the QTLs

In the present study, a total of 101 linkage based mapping studies were used, in which different types of mapping populations were utilized for mapping including: recombinant inbred lines (RILs), doubled haploid (DH), and F_2:3_ or F_3_ populations. The information on as many as 505 QTLs was compiled from these 101 studies (Supplementary Table 1). The detailed information on these QTLs involving the chromosomes, flanking markers, genetic positions, PVE values and LOD scores for individual QTLs is presented in Supplementary Table 1. The important characteristics of the collected QTLs were: (i) whole genome distribution with number of QTLs mapped per chromosome varied from 5 on chromosome 6D to 67 on chromosome 2B (Fig. 1a); (ii) differential distribution of QTLs among the three sub-genomes, with 142 (28.11% of total) QTLs on the A sub-genome, 288 QTLs (57.02%) on the B sub-genome and 75 QTLs (14.85%) on the D sub-genome; and (iii) the number of QTLs for twelve individual parameters relevant to stripe rust ranged from 1 QTL for IR to 216 for DS. Furthermore, 186 QTLs were associated with more than two parameters each, and 18 QTLs with more than three parameters each (iv) LOD scores for individual QTLs ranged from 2.3 to 35.55, with as many as 268 QTLs (53.06% of the total) possessing LOD value of < 6 (Fig. 1b), (v) the PVE% by an individual QTLs varied from 1 to 88% and displaying a typical L-shaped distribution, with most (46.33%) QTLs showing a PVE < 10% and only a small fraction (9.10%) representing major QTLs/genes (PVE > 40%) (Fig. 1c).

### Consensus map and projection of QTLs

The high density “WheatConsensusMap-2022” that was used during the present study showed huge variation for the genetic lengths of the maps of individual chromosomes (the length varied from 294.84 cM for chromosome 4D to 716.95 cM for chromosome 5A, with an average map length of 509.72 cM). The number of different molecular markers mapped on an individual chromosome varied from few hundred (367 on 4D) to several thousands (18,990 on 3B), with an average of approximately 6,598 markers per chromosome. The total map length of all the chromosomes in the consensus map was 10,704.2 cM covered by 138,574 markers. Because the consensus map was constructed using distinct genetic maps with various numbers and types of markers, the distribution of markers at the two ends differed significantly, with higher marker density was found at the chromosome fore-ends. The marker density on an individual wheat chromosome varied from 1.24 markers per cM on chromosome 4D to 30.41 markers per cM on the largest wheat chromosome 3B, with an average of 12.95 markers per cM across the whole genome.

Further, a total of 380 QTLs (75.24% of the total QTLs) collected from the literature were projected on to this newly developed highly dense consensus map. The remaining 101 QTLs could not be projected onto the consensus map owing to either of the reasons discussed in one of our earlier study [43].

### MQTLs for stripe rust resistance

**Fig. 2:**
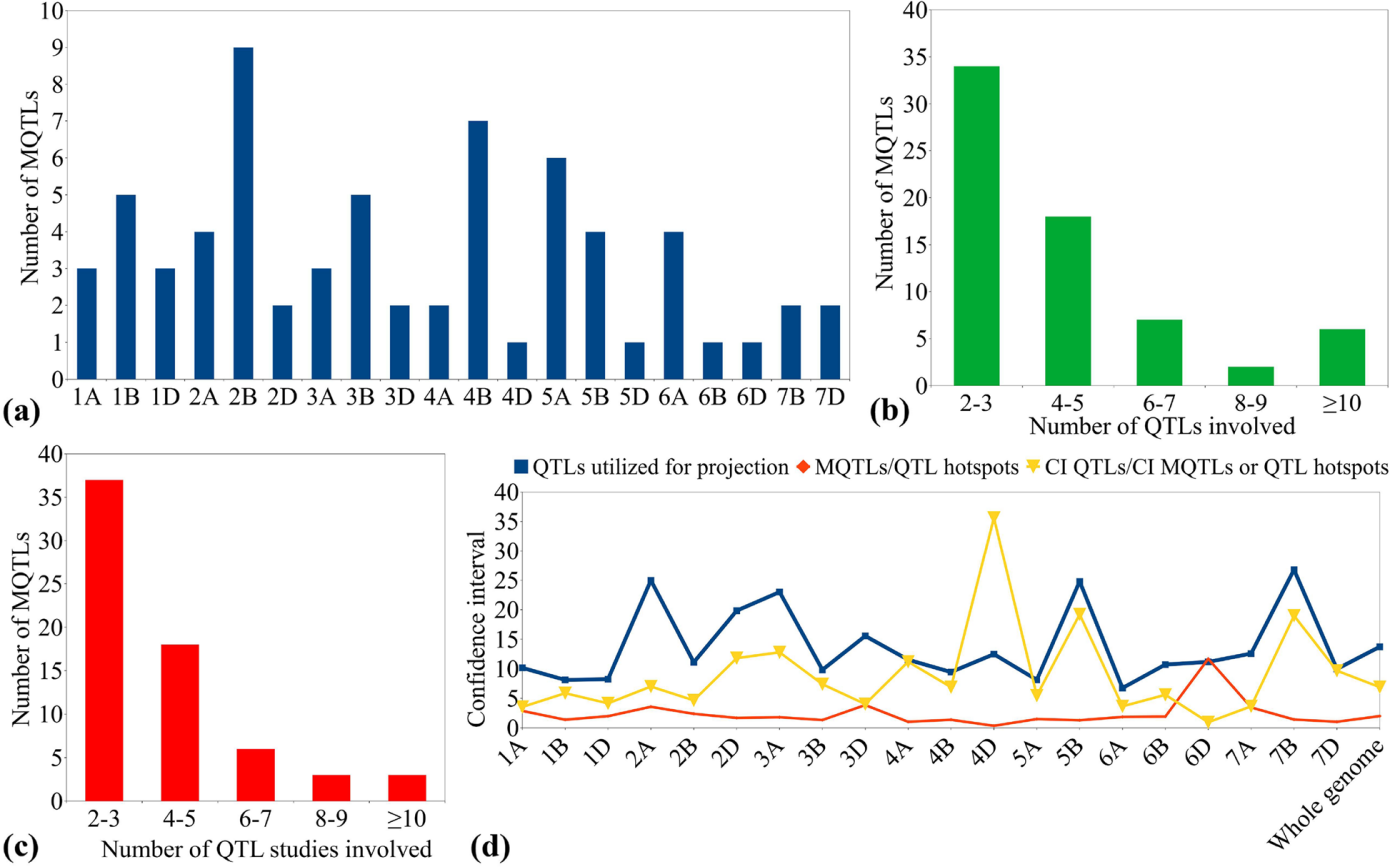
Basic characteristics of MQTLs associated with yellow rust resistance (**a**) chromosome wise distribution of MQTLs, (b) the number of QTLs involved in different MQTLs, (**c**) the number of QTL studies involved in different MQTLs, (**d**) fold reduction in confidence intervals of QTLs after meta-analysis

The Veyrieras approach [38] was used for meta-analysis of QTLs available on all wheat chromosomes except for 1D, 4D, 5D, 6D and 7A as these chromosomes had < 10 projected QTLs per chromosome and therefore the Goffinet and Gerber’s approach [39] was used for analyzing the QTLs on these chromosomes. Overall, a total of 67 MQTLs were predicted for stripe rust resistance (Fig. 2a, Table 1, Supplementary Table 2), which were based on 309 initial QTLs out of 380 projected QTLs. The remaining 71 QTLs included 24 singletons **(**Supplementary Table 3), 43 QTLs with peaks outside the supporting intervals of MQTLs and 2 QTL hotspots (each involving 2 initial QTLs from a single study) (Supplementary Table 4). The 24 singletons were mapped on wheat chromosomes 1A, 2A, 2B, 3A, 3B, 3D, 4A, 4B, 4D, 5D, 6A, 6D, and 7A, and 2 QTL hotspots were mapped on chromosomes 5D and 7A.

**Table 1:** MQTLs associated with stripe rust resistance detected during the present study

MQTL name	CI (cM)	Flanking markers	N QTLs (N studies)	LOD score (PVE, %)	Component traits
MQTL1A.1	150.41–153.18	Kukri_rep_c81545_195/IAAV2838	3 (3)	5.3 (14.83)	FDS, DS/IT, DS
MQTL1A.2	154.09–155.31	IAAV2838/IAAV2838	3 (3)	5.63 (8.43)	DS, DS/IT, AUDPC/IT/SR/LAI
MQTL1A.3	177.32–181.96	wsnp_Ku_c34659_43981982/IWB69234	2 (2)	6.35 (12.15)	DS, IT/DS
MQTL1B.1	94.37–95.30	BS00095286_51/Kukri_c7393_285	5 (5)	12.65 (25.12)	IT/DS (2), IT, DS, AUDPC/IT
MQTL1B.2	145.25–146.93	GENE-1756_115/wsnp_Ex_c14273_22230844	5 (5)	11.08 (16.204)	DS (3), IT, AUDPC/IT
MQTL1B.3	153.68–155.15	wsnp_Ex_c14273_22230844/RFL_Contig4873_542	6 (4)	9.75 (11.18)	DS/IT (2), DS (3), IT
MQTL1B.4	198.58–200.21	BobWhite_c4147_1351/BS00066135_51	3 (3)	6 (10.43)	SR, DS/AUDPC, DS
MQTL1B.5	233.79–235.01	IWB70151/Excalibur_rep_c107001_320	4 (4)	11.81 (23.20)	AUDPC/IT, DS (2), IT/DS
MQTL1D.1	35.08–37.54	wsnp_Ra_c48124_53475145/AX-110276692	2 (2)	8 (21.25)	DS, IT/DS
MQTL1D.2	72.05–72.75	Kukri_c46169_294/AX-109701841	2 (2)	2.94 (1.69)	DS (2)
MQTL1D.3	74.97–77.75	Kukri_c20446_215/AX-110163017	2 (2)	8.55 (9.49)	DS (2)
MQTL2A.1	185.38–185.53	BS00004724_51/wsnp_Ex_rep_c103167_88181968	11 (9)	13.38 (21.18)	AUDPC, DS (6), IT, IT/DS, RT/DS, SR
MQTL2A.2	210.34–214.06	D_GA8KES401BVP4P_43/IAAV2585	2 (2)	13.55 (21.15)	DS (2)
MQTL2A.3	278.26–287.31	BobWhite_c1611_1685/BS00078612_51	3 (3)	12.49 (34.63)	DS/SR, DS/AUDPC, IT
MQTL2A.4	332.98–334.34	AX-110595053/IWB6967	2 (2)	15.525 (13.15)	DS, IT/DS
MQTL2B.1	29.97–32.74	Ku_c2441_1342/BobWhite_c20346_138	4 (4)	4.8975 (19.275)	AUDPC/IT (2), IT, DS
MQTL2B.2	54.87–60.28	RAC875_c8069_1709/Excalibur_c4372_262	2 (2)	4.82 (10.17)	IT, DS/SR
MQTL2B.3	60.96–63.43	BS00066389_51/Excalibur_c39493_251	2 (2)	35.55 (56.9)	DS/IT, DS
MQTL2B.4	100.57–101.86	Kukri_c94792_127/Jagger_c10188_98	11 (9)	12.26 (26.26)	DS/IT, AUDPC/IT/SR/LAI, DS (7), AUDPC/IT, IT/SN
MQTL2B.5	102.84–103.56	RAC875_c16993_196/BS00070050_51	11 (9)	12.43 (26.95)	DS (4), DS/IT, IT/SN (2), RT/DS, AUDPC, FDS/NDVI, DS/RT/NDVI
MQTL2B.6	110.17–110.90	BS00049876_51/Ku_c68139_836	4 (4)	25.16 (36.19)	AUDPC, DS/AUDPC, AUDPC/IT, IT/DS
MQTL2B.7	120.49–122.42	IWB8416/BS00072620_51	2 (2)	8.10 (13.85)	DS, AUDPC/DS
MQTL2B.8	140.21–143.45	AX-94718406/IWB67084	5 (3)	4.61 (7.822)	DS (5)
MQTL2B.9	199.06–201.96	IWB62599/BobWhite_c31708_99	2 (2)	8.33 (18.9)	DS, IT
MQTL2D.1	75.42–78.47	AX-94898597/AX-95176044	2 (2)	13.45 (38.4)	IT, LP/IT
MQTL2D.2	98.67–98.98	RAC875_c11911_431/D_contig28346_467	9 (6)	5.98 (13.75)	IT, DS (5), AUDPC/DS, AUDPC
MQTL3A.1	49.57–51.12	Excalibur_c32653_553/BS00109084_51	2 (2)	4.66 (14.325)	DS/IT, AUDPC/IT
MQTL3A.2	125.99–128.88	3A_s3948706//wsnp_Ra_c9738_16174002/Tdurum_contig75764_146	4 (3)	5.08 (12.27)	SR (2), DS (2)
MQTL3A.3	142.47–143.43	Excalibur_c20448_318/Excalibur_c98205_83	3 (3)	4.53 (8.33)	LP/IT, DS (2)
MQTL3B.1	17.01–18.19	Excalibur_c20277_436/RAC875_rep_c111781_146	3 (3)	8.3 (14.37)	IT/AUDPC, AUDPC/DS, DS
MQTL3B.2	29.78–34.05	BS00011904_51/RAC875_c30148_90	4 (4)	6.1 (10.025)	SR, DS (2), IT/AUDPC
MQTL3B.3	73.84–74.22	BS00075373_51/Tdurum_contig14251_431	16 (11)	8.13 (13.10)	IT (3), SR, DS/IT (5), DS (4), DS/SR (2), AUDPC
MQTL3B.4	74.53–75.31	BS00075373_51/Tdurum_contig14251_431	4 (4)	22.47 (29.18)	RT/DS, IT/DS, DS, IT/DS/AUDPC
MQTL3B.5	92.80–92.83	IWB47273/AX-111655083	9 (7)	6.57 (18.11)	IT/DS/AUDPC, DS (3), DS/SR (2)
MQTL3D.1	77.87–83.56	BS00021930_51 /wPt-3815//AX-108885897	3 (2)	6.4 (10.4)	IT/DS, DS (2)
MQTL3D.2	117.24–119.28	IAAV5635/wPt-666738//IAAV2827	2 (2)	9.2 (12.2)	IT/DS
MQTL4A.1	133.66–134.57	wsnp_Ex_c4752_8482625/AX-108737352	5 (4)	5.65 ((12.18)	DS (3), DS/AUDPC, IT
MQTL4A.2	181.44–182.59	Xiwa2606//IWB27365/SBG_177633//IWA3068	5 (4)	18.18 (16.44)	AUDPC/IT/SR/LAI (2), DS/RT/NDVI, SR, LAI/RT
MQTL4B.1	32.7–34.42	Tdurum_contig61142_146/Ra_c26080_461	4 (4)	4.65 (7.54)	DS (3), DS/IT
MQTL4B.2	52.99–53.38	Tdurum_contig94552_326/Tdurum_contig13165_443	4 (4)	5.13 (6.51)	DS/IR, DS (2), AUDPC
MQTL4B.3	59.74–60.63	Tdurum_contig29989_132/BS00067786_51	4 (4)	11.72 (27.25)	DS (3), DS/IT
MQTL4B.4	62.63–64.06	BS00063804_51/IAAV5175	3 (3)	7.57 (16.77)	DS/IT (2), DS
MQTL4B.5	83.5–86.3	AX-109899078/IWB7491	2 (2)	4.35 (19.85)	DS, AUDPC
MQTL4B.6	104.8–105.74	CAP12_c4704_232/RAC875_c6694_906	2 (2)	15.03 (27.11)	DS, IT/DS/AUDPC
MQTL4B.7	116.01–117.44	IWB46525/Kukri_c18722_425	3 (3)	8.67 (21.87)	DS/SR (2), AUDPC/IT
MQTL4D	93.32–93.67	Kukri_rep_c68594_530/RFL_Contig2917_500	3 (3)	4.19 (5.66)	DS (3)
MQTL5A.1	230.77–232.75	IAAV2080/GENE-3572_70	5 (5)	5.22 (7.46)	AUDPC/IT (2), DS (3)
MQTL5A.2	243–243.86	BobWhite_c17440_130/BS00022110_51	2 (2)	8.9 (4.25)	IT/DS, DS/AUDPC
MQTL5A.3	252.68–254.08	Ex_c6161_335/IWB56891	2 (2)	3.29 (1.88)	DS (2)
MQTL5A.4	262.77–263.05	Xbarc100/IWB31441	4 (4)	9.92 (6.89)	AUDPC/DS, DS, SR, IT/DS
MQTL5A.5	314.26–317.34	BS00085826_51/CRA-4160//IWB14724	4 (4)	4.27 (6.49)	DS (3), IT/DS/AUDPC
MQTL5A.6	372.08–373.41	wsnp_Ex_c18941_27840933/Kukri_c34193_102	2 (2)	22.82 (35.52)	DS, AUDPC/IT
MQTL5B.1	3.165–5.375	IWB65830/wsnp_Ex_c6100_10676217	7 (6)	8.27 (7.99)	DS (4), IT (3)
MQTL5B.2	46.86–47.9	Xgwm544//IWB21416/IWB32919	7 (7)	5.61 (13.68)	SR, DS (3), AUDPC/IT, AUDPC, IT
MQTL5B.3	106.37–108.11	Tdurum_contig5522_455/BobWhite_c15406_510	6 (5)	12.01 (12.96)	DS (4), DS/IT (2)
MQTL5B.4	138.72–138.92	BS00024829_51/RAC875_c14078_202	4 (3)	4.5 (12.02)	SR, AUDPC/IT (2), IT/DS
MQTL5D	301.76–303.55	Excalibur_rep_c73156_287/BS00021911_51	2 (2)	4.62 (8.37)	IT/DS, AUDPC/IT
MQTL6A.1	176.27–178.23	Kukri_c4606_620/BS00084846_51	7 (6)	7.20 (9.03)	DS (4), AUDPC/IT/SR/LAI, IT, LP/IT
MQTL6A.2	186.3–188.46	BS00041481_51/BS00072146_51	2 (2)	8.97 (27.39)	IT/DS, IT
MQTL6A.3	199.27–199.95	BS00064548_51/BS00037006_51	3 (3)	4.47 (7.47)	AUDPC, AUDPC/DS, DS/IT
MQTL6A.4	219.29–222.16	wsnp_RFL_Contig3136_3092151/Kukri_c62719_188	2 (2)	8.25 (19.45)	IT/AUDPC, DS/IT
MQTL6B	106.76–106.76	wPt-5176/Xgwm193	24 (20)	9.23 (17.24)	IT/DS (4), AUDPC/IT/SR/LAI, DS (6), IT/AUDPC (3), IT/DS/AUDPC, DS/AUDPC, SR (3), AUDPC (4), IT
MQTL6D	164.26–175.94	D_GDRF1KQ02FFPXT_243/Xpsr8//Kukri_c25717_133	2 (2)	2.43 (13.1)	AUDPC/IT
MQTL7B.1	116.89–119.52	Excalibur_c12500_116/Ra_c39042_935	14 (12)	3.82 (6.86)	LP/IT, DS (5), SR, AUDPC (2), DS/SR, IT (2), FDS
MQTL7B.2	139.91–140.09	IAAV5530/BobWhite_c44404_312	3 (3)	3.34 (9.81)	IT, DS/SR, IT
MQTL7D.1	55.24–56.97	Excalibur_c30913_512/BS00027514_51	6 (5)	11.46 (23.43)	DS (4), DS/IT, DS/AUDPC
MQTL7D.2	67.54–67.87	D_contig66049_34/Kukri_c37802_1215	7 (7)	16.71 (23.43)	AUDPC, SR, AUDPC/IT/SR/LAI, DS (3), AUDPC/IT

*IR* Infection rate, *DS* Disease severity, *FDS* Final disease severity, *AUDPC* Area under disease progress curve, *IT* Infection type, *SR* Stripe rust response, *NDVI* Normalized difference vegetation index, *LP* Latency period, *RT* Reaction type, *IR* Infection response, *LAI* Leaf area infected, *SN* Number of stripes per 10 cm^2^ leaf area

A total of 22 MQTLs were predicted on sub-genome A, with the highest number of MQTLs (6) on chromosome 5A and 4 MQTLs each on chromosomes 2A and 6A, while chromosomes 1A and 3A each harbored 3 MQTLs, and chromosome 4A contained only 2 MQTLs. For B sub-genome, a total of 33 MQTLs were predicted, representing the sub-genome with the highest number of MQTLs. Chromosome 2B was observed to have 9 MQTLs which is the highest number of MQTLs on a chromosome of this sub-genome, followed by 7 MQTLs on chromosomes 4B, 5 MQTLs each on chromosomes 1B and 3B, and 4 MQTLs on 5B, whereas, chromosomes 6B and 7B had the lowest number of MQTLs (1 and 2 MQTLs, respectively). Similarly, a total of 12 MQTLs were predicted on sub-genome D, with chromosome 1D harboring the maximum 3 MQTLs, followed by 2 MQTLs each on chromosomes 2D, 3D, and 7D, whereas each of the chromosomes 4D, 5D, and 6D contained only a single MQTL (Fig. 2a). The number of QTLs per MQTL varied from ≤ 3 in 34 MQTLs to > 10 QTLs in the six MQTLs (viz., MQTL2A.1, MQTL2B.4, MQTL2B.5, MQTL3B.3, MQTL6B, and MQTL7B.1) (Fig. 2b), whereas the number of QTL studies involved in individual MQTLs ranged from ≤ 3 in 37 MQTLs to ≥ 5 in 17 MQTLs (Fig. 2c).

**Fig. 3:**
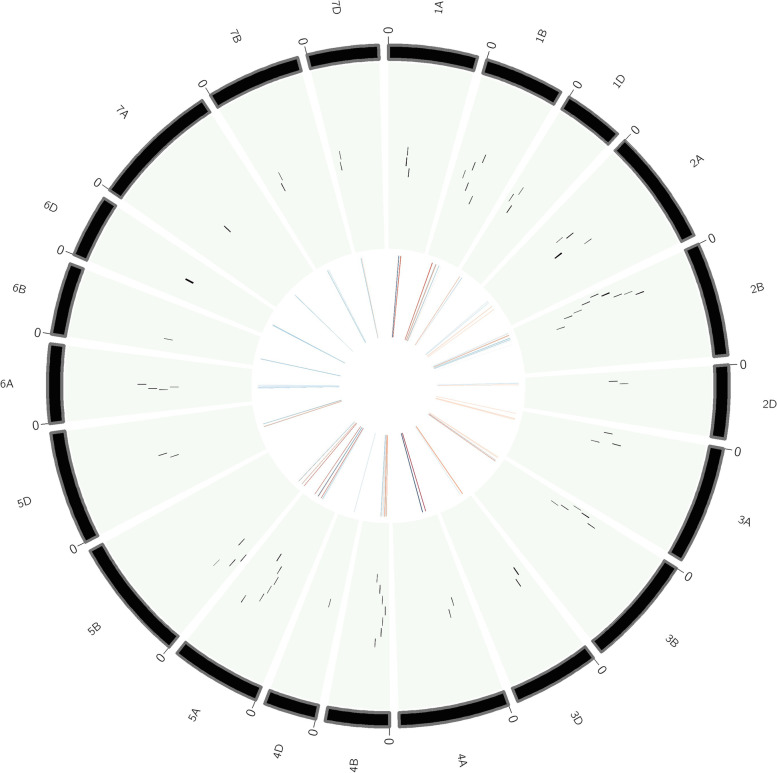
Circular diagram representing the features of QTLs and MQTLs associated with yellow rust resistance. The information projected includes, (moving inwards) the outermost ring represents consensus map, the positions of MQTLs on the chromosomes, and the innermost ring represents the frequency of QTLs involved in each identified MQTL

**Fig. 4:**
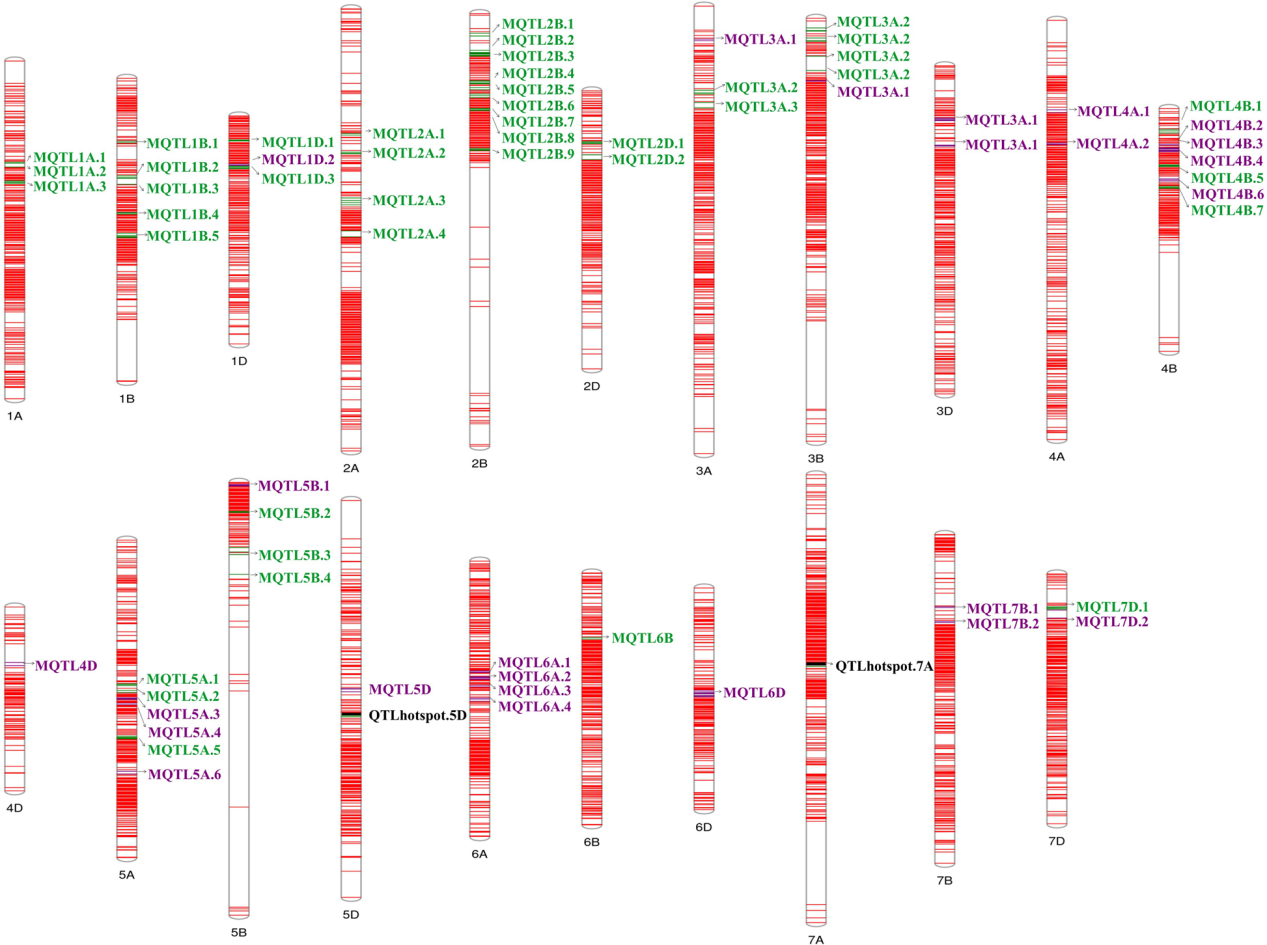
Distribution of MQTLs on different wheat chromosomes; MQTLs: green, GWAS validated MQTLs: purple, and QTL hotspots: black

For the reported MQTLs and QTL hotspots, the CI varied from 0 to 11.68 cM, with an average of 1.97 cM (Fig. 2d). The average CI of MQTLs and QTL hotspots was 6.89-fold less than that of original QTLs, and there were substantial differences in the CI reduction among different wheat chromosomes. The average CI on chromosomes 4D and 5B was reduced by 35.62 and 19.21-fold, respectively, followed by 18.99 and 12.78-fold on chromosomes 7B and 3A, respectively. The available 67 MQTLs were also physically anchored on the wheat reference genome. The mean physical CI of the MQTLs was 24.01 Mb, which ranged from 0.0749 (MQTL2A.2) to 216.23 Mb (MQTL5B.2). The PVE (%) of reported MQTLs ranged from a minimum of 1.69 to a maximum of 56.90, and the LOD score ranged from 2.43 to 35.55. The average PVE and the LOD score of the MQTLs were 16.28% and 9.11, respectively. Salient characteristics exhibited by the initial QTLs, MQTLs and their distribution on different wheat chromosomes are shown in (Figs. 3 and 4), respectively.

### Validation of MQTLs through genome-wide association studies

The physical coordinates of the 67 MQTLs were also compared with marker-trait associations (MTAs) reported in 20 earlier GWAS conducted for stripe rust resistance. Out of these 67 MQTLs, 44 could be validated (Fig. 4), in at least one GWAS (involving a total of 297 MTAs) (Supplementary Table 5). In addition, 11 and 5 MQTLs could also be verified using MTAs associated with leaf and stem rust resistance, respectively, in addition to stripe rust resistance. The number of MTAs co-localized with an individual MQTL also varied. It was noticed that as many as 15 MQTLs could found correspondence with at least 2 MTAs; of these, MQTL1B.4 matched with 40 MTAs detected in 3 GWAS, followed by MQTL1B.2 and 1B.3 each with 36 MTAs identified in 3 GWAS and MQTL1B.1 with 14 MTAs detected in 4 GWAS.

### *Yr* gene co-localization with MQTLs

The study of the association of *Yr* genes with individual MQTLs revealed that a total of ten stripe rust resistance genes, including *Yr5*, *Yr7*, *Yr16*, *Yr26*, *Yr30, Yr43*, *Yr44, YrCH52*, *YrH52* and *Yr64,* were found to be co-localized with fourteen MQTLs predicted in this study (Supplementary Table 6). For instance, MQTL1B.5 co-localized with *YrH52,* and MQTL2D.2 co-localized with *Yr16*. Due to the overlapping nature of a few MQTLs, some genes were co-localized with more than one MQTL; for instance, MQTL3B.1, MQTL3B.2, MQTL3B.3, and MQTL3B.4 each co-localized with *Yr30* and MQTL1B.2 and MQTL1B.3 each co-localized with *Yr64*. On the other hand, there were also a few MQTLs that co-localized with more than one *Yr* genes. For instance, MQTL2B.6 co-localized with both *Yr5* and *Yr7*, MQTL2B.9 co-localized with both *Yr43* and *Yr44*, and MQTL1B.4 co-localized with both *Yr26* and *YrCH52,* conferring adult plant resistance.

### Candidate genes identified from the high confidence MQTLs (hcMQTLs) and their expression in different wheat tissues

To further improve the quality of the MQTLs predicted, they were further refined to regions termed as high-confidence MQTLs (hcMQTLs). The hcMQTLs consist of 29 consensus regions with an average genetic CI and physical CI of 1.26 cM and 3.51 Mb, respectively. Overall, each hcMQTL cluster contained at least three initial QTLs. The B genome had 17 hcMQTLs which is the highest number of hcMQTLs in a sub-genome. Within the B sub-genome, chromosome 3B contained the highest 5hcMQTLs. In addition, hcMQTL2B.5 had the smallest physical interval (0.12 Mb), while hcMQTL3B.1 had the largest interval, covering 13.28 Mb. Subsequently, (CG) mining within hcMQTLs revealed 1,562 unique gene models (299 duplicated genes available from overlapping regions were removed), with hcMQTL3B.1 possessing the highest number of genes (269) and hcMQTL3A.2 possessing the lowest number (4) of genes (Supplementary Table 7).

Further, the expression analysis with ERP009837 dataset revealed 30 differentially expressed candidate genes (DECGs), whereas, with the second dataset, ERP013983, revealed a total of 103 DECGs. Overall, a total of 123 genes were found to be differentially expressed across the two expression datasets utilized (Supplementary Table 8). Fifty-nine most promising CGs were chosen from the 123 DECGs, which encode proteins belonging to different classes, such as (i) R-domain containing proteins including protein kinases, (ii) transcription factors (TFs) such as WRKY-TF and zinc finger TF, (iii) proteins that participate in oxidation–reduction reactions such as cytochrome P450, (iv) cupin superfamily proteins such as germin-like protein, (v) glycosyltransferase enzymes such as UDP-glucuronosyl/UDP-glucosyltransferase, and (vi) WD40 repeat containing proteins.

In addition, the expression profiles of all 59 DECGs could be examined in various wheat tissues and during various development stages. The 59 DECGs could be clustered into two classes based on their patterns of expressions in various tissues (Supplementary Fig. 1). Genes in Class I exhibited moderate to high expression in the fifth leaf blade and flag leaf blade at the flowering stage in comparison to the other stages of plant development. Some of the Class I genes are as follows: *TraesCS1B02G020600* (hcMQTL1B.1), *TraesCS1B02G020700* (hcMQTL1B.1), and *TraesCS3B02G024500* (hcMQTL3B.1). In addition, some genes were significantly expressed in the first leaf sheath at the tillering stage. Some of the Class II genes are as follows: *TraesCS3A02G046000* (hcMQTL3A.3), *TraesCS3A02G046100* (hcMQTL3A.3), *TraesCS3B02G037000* (hcMQTL3B.1)*, TraesCS3B02G023700* (hcMQTL3B.1)*, TraesCS4B02G041100* (hcMQTL4B.1)*,* and *TraesCS4D02G031800* (hcMQTL4D.1)*.*

## Discussion

The genetics of quantitative resistance to stripe rust has been explored in numerous cultivars of wheat using improved QTL mapping methodologies (http://www.wheatqtldb.net/). This has led to the identification of a large number of QTLs (> 500 QTLs) associated with stripe rust resistance in wheat (Supplementary Table 1). It is a normal phenomenon that QTLs discovered using one mapping population does not truly work well in a breeding program that involves a different population/parental lines [55]. Hence, there was an urgent need to reanalyze the already identified QTLs through meta-analysis of QTLs, which is one of the most promising methodologies for the integration of QTLs and the prediction of stable and robust MQTLs that are frequently involved in trait variation and can addresses the heterogeneity that exists between studies [37, 43].

Overall, meta-analysis combines data from several QTL mapping studies in diverse environments and different genetic backgrounds to identify stable, major and reliable MQTLs with reduced CIs [56]. MQTL analysis has already been conducted for a large number of traits, including disease resistance, in different crops, such as rice [57, 58], wheat [45, 51, 52, 59, 60], and maize [61]. Most recently, MQTLs were predicted for multiple disease resistance [59], where MQTLs were predicted for all three rusts utilizing a comparatively small number of initial QTLs associated with stripe rust resistance, and very few MQTLs associated with only stripe rust resistance were identified. The accuracy of the results of the meta-analysis is generally positively correlated with the number of initial QTLs utilized.

Therefore, during the present investigation, MQTL analysis was performed based on QTLs (published so far) conferring stripe rust resistance collected from various independent experiments to acquire a better understanding of the regulation of stripe rust resistance in wheat. The projection of the original QTLs onto a consensus map is the first stage in determining consensus regions using meta-analysis. The B sub-genome showed the highest marker saturation and thus carried the maximum number of QTLs, which is consistent with earlier studies characterizing genetic diversity and unraveling complex genetic architecture of disease resistance in wheat [45, 46, 59, 60]. The low level of polymorphism associated with sub-genome D could be one explanation for the small number of QTLs found on sub-genome D across the different QTL mapping studies which is in accordance of previous meta-analyses for disease resistance in wheat showing smaller number of QTLs on sub-genome D [48, 51, 52, 59, 60]

We believe that this is the most comprehensive collection and meta-analysis of QTL for stripe resistance in wheat to date. The entirety of the QTLs here analyzed can be referred to as the “QTLome” [62] of stripe rust resistance in wheat, as it brings together most of the loci mapped in the crop until now in a global analysis. In the present study, a larger number of QTLs (75.24%) were projected onto the consensus map compared with the fewer number of QTLs projected in the previous MQTL analyses for leaf rust [45, 46]. A possible reason could be the utilization of a dense consensus map during the present study. The identification of 67 MQTLs from 380 QTLs resulted in a 5.67-fold (380/67) reduction in the number of genomic regions or QTLs associated with stripe rust resistance in wheat. However, the reductions of 4.11-fold and 4.27-fold were observed in previous studies while conducting MQTL analyses for leaf rust resistance in wheat [45, 46].

Our study is far more updated and comprehensive, and it differs from the previous meta-analysis study conducted for stripe rust resistance [54] in several aspects. Some of the major differences between the previous study and the current study in terms of data used and results obtained are as follows- (i) the current study used 505 QTLs that were collected from 101 mapping studies, in contrast to the earlier study [54], which used only 353 QTLs that were collected from just 75 studies. The number of initial QTLs utilized for meta-QTL analysis has been found to be significantly and positively correlated with the accuracy of the statistical findings [43, 44]; (ii) in contrast to the earlier study, where a consensus map was created using only 76,753 markers [54], the current study involved a dense consensus map involving 138,574 markers; (iii) in contrast to the previous study, where only 184 QTLs could be grouped into the MQTLs, the use of a dense consensus map during the present study enabled the inclusion of an increased number of QTLs (309 QTLs) into MQTLs; (iv) further, as many as 44 MQTLs predicted during the present study were validated with MTAs available from GWAS in contrast to the earlier study where no such efforts for validating MQTLs were made [54]. Validation of MQTLs with GWAS-based MTAs suggests that the impact of these genomic regions on stripe rust resistance may be less limited by genetic background; (v) we observed an average CI of MQTLs of 1.97 cM and a 6.89-fold reduction in CI of initial QTLs after meta-analysis in the current study; no such statistics were reported in the previous study [54]; (vi) we observed significant reduction (i.e., 5.67 fold) in the number of genomic regions associated with stripe rust resistance after meta-analysis, this is in contrast to earlier study [54], where only 3.5-fold reduction was observed; (vii) rather than analyzing all available MQTLs (irrespective of their importance) for CGs as done in previous study [54], we used a set of criteria to prioritize some hcMQTLs for CG mining, which enabled the identification of promising CGs; and (viii) in the current study, we examined the patterns of important genes (showing differential expression under disease infection) in wheat tissues at various developmental stages. This analysis allowed us to distinguish CGs predicted to be associated with seedling and adult plant resistance. A detailed comparison of both studies is provided in the Supplementary Table 9.

Even after several years of research in the search for QTLs related to stripe resistance in wheat, the reports continue to publish at an astounding rate. For example, 11 new mapping studies have been published during the final writing and the peer-review period of this article [63–73] reporting the identification of as many as 83 QTLs associated with different parameters of stripe rust resistance in wheat. These studies could not be included in the main analysis, but we compiled information on these new QTLs (Supplementary Table 10) to provide researchers with up-to-date information on QTLs associated with stripe rust resistance for use in other genetic and basic studies, such as QTL fine mapping and cloning. Furthermore, to determine whether they are novel QTLs or just parts of MQTLs identified in this study, we performed a preliminary analysis by comparing the physical positions of the QTLs with the physical coordinates of MQTLs. As many as 13 QTLs were found to overlap with 19 MQTLs on the following six chromosomes: 2B, 3B, 4B, 5A, 5B, and 6A (Supplementary Table 10).

Further, most of the MQTLs identified in the present study governed numerous component traits/parameters indicating either the existence of pleiotropic genes or a close interaction between genes for distinct parameters. This may be attributable to a bias in the detection of stripe rust resistance due to the inclusion of associated traits, as previously demonstrated in the MQTL study of leaf rust resistance in wheat [46]. In the present study, 65.67% of the MQTLs could be validated with earlier GWAS reports. Furthermore, the earlier reports of validation of MQTLs with GWAS include results that are similar to the results of the present study, with 60.31% and 62.79% MQTLs validated with GWAS [59, 60]. These widely different results may be attributed to the nature of materials used for interval mapping (subsequently utilized for meta-analysis) and GWAS [28].

### Co-localization of MQTLs with stripe rust resistance genes

A search for co-localization of stripe rust resistance genes and MQTLs was conducted to support the location of MQTLs discovered in the present investigation. As per the literature, 83 stripe rust resistance genes have been mapped and documented in wheat (https://shigen.nig.ac.jp/wheat/komugi/genes/symbolClassListAction.do?geneClassificationId=222), and a few of them have also been cloned and identified to encodes proteins for NBS-LRR and represents “R genes” [74, 75]. A total of ten stripe rust resistance genes (viz., *Yr5*, *Yr7*, *Yr16*, *Yr26*, *Yr30, Yr43*, *Yr44, YrCH52*, *YrH52* and *Yr64*) were found to be co-localized with fourteen MQTLs; for instance, *Yr26*, *Yr5* and *Yr30* co-localized with MQTL1B.4, MQTL2B.6 and MQTL3B.1 on chromosomes 1B, 2B, and 3B, respectively. Occasionally, a single *Yr *gene is linked to multiple MQTLs, notably on chromosomes 1B and 3B due to overlapping of physical intervals of a few MQTLs. Further, MQTL1B.4, co-localized with two stripe rust genes (*Yr26* and *YrCH52*) conferring all-stage resistance to stripe rust in wheat, thus confirming the usefulness of using saturated consensus maps for MQTL analysis.

Law [76] reported the location of *Yr7* on chromosome 2BL, and McIntosh [77] showed its close association with the stem rust resistance gene *Sr9g*. In addition, linkage studies on the stripe rust resistance gene *YrSp* [78], which is also located on the long arm of chromosome 2B, with *Yr5* and *Yr7* demonstrated that *YrSp* is also allelic to both *Yr5* and *Yr7* [79]. Both genes confer all-stage resistance or seedling resistance and are thought to have evolved from *Triticum aestivum* L. subsp. spelt a (L.). However, in this study, MQTL2B.6 co-localized with both the genes *Yr5* and *Yr7*. Additionally, the gene *Yr43* resistance is race specific, located on chromosome 2BL, and should be used in combination with other genes or QTLs for either all-stage resistance or durable HTAP resistance. Cheng and Chen [80] reported that *Yr43* is linked to another stripe rust resistance gene, previously identified as *YrZak*, in cultivar Zak, later designated as *Yr44*. In this study, MQTL2B.9 was found to be co-localized with both genes (*Yr43* and *Yr44*), indicating an association between them.

### Candidate genes within the hcMQTLs and their association with stripe rust resistance

Candidate gene mining within 29 hcMQTLs revealed 1,562 unique gene models, which also included 123 DECGs. Fifty-nine promising CGs were chosen from these 123 DECGs based on the information available from the literature (Table 2). The roles of some of these genes in providing resistance to stripe rust may be discussed as follows: (i) NBS-LRR domain-containing genes encode the proteins that are also encoded by cloned *Yr* genes, such as *Yr5* and *Yr10*, as mentioned earlier [74, 75]. (ii) Protein kinase family proteins play a crucial role in enhancing disease resistance in wheat. Receptor-like kinases (RLKs) and plant protection kinases regulate the detection and activation of a wide range of developmental and physiological signals, including those related to defense and symbiosis [81, 82]. (iii) The wheat WRKY TFs, *TaWRKY49* and *TaWRKY62* are responsible for differential HT seedling-plant resistance to stripe rust in wheat. (iv) Roles of different zinc finger-containing proteins have been widely discussed in the literature [83]; furthermore, it has also been inferred that the presence of zinc finger domains, in combination with NBS-LRR domains in resistance proteins, can reflect a major function of these domains in host–pathogen interactions [83]. (v) The gene *TaCYP72A* encoding cytochrome P450 was found to contribute to host resistance against *Fusarium* head blight in wheat [84]. (vi) Plant glycoproteins known as germin-like proteins (GLPs) are water-soluble and are members of the cupin superfamily. It is well known that GLPs play a significant role in how plants react to various abiotic and biotic stresses, including pathogens [85]. (vii) A gene encoding WD40-repeat protein was reported to function in a histone deacetylase complex to fine-tune defense responses to powdery mildew in wheat [86].

**Table 2:** Most promising candidate genes associated with stripe rust resistance

hcMQTL	Gene	Position (bp)	Function description
MQTL1A.1	TraesCS1A02G040400	22,287,727–22,291,553	RNA recognition motif domain
MQTL1A.1	TraesCS1A02G041400	22,656,561–22,657,221	Proteinase inhibitor I13, potato inhibitor I
MQTL1B.1	TraesCS1B02G020600	9,592,538–9,599,744	Protein kinase domain
MQTL1B.1	TraesCS1B02G020700	9,592,944–9,596,685	Protein kinase domain
MQTL2B.1	TraesCS2B02G010500	5,684,671–5,689,444	WRKY domain
MQTL2B.1	TraesCS2B02G012000	6,178,676–6,180,145	UDP-glucuronosyl/UDP-glucosyltransferase
MQTL2B.4	TraesCS2B02G603800	785,885,897–785,899,606	Pentatricopeptide repeat
MQTL2B.5	TraesCS2B02G605000	786,229,219–786,233,560	C2 domain
MQTL3A.2	TraesCS3A02G043800	23,830,054–23,831,765	ABA DEFICIENT 4-like
MQTL3A.3	TraesCS3A02G046000	24,611,605–24,612,579	Proteinase inhibitor I12, Bowman-Birk
MQTL3A.3	TraesCS3A02G046100	24,678,221–24,679,061	Proteinase inhibitor I12, Bowman-Birk
MQTL3A.3	TraesCS3A02G046300	24,748,236–24,748,973	Proteinase inhibitor I12, Bowman-Birk
MQTL3B.1	TraesCS3B02G012700	5,789,746–5,790,925	Protein kinase domain
MQTL3B.1	TraesCS3B02G015000	6,294,081–6,303,451	WD40 repeat
MQTL3B.1	TraesCS3B02G015400	6,390,297–6,393,323	Protein kinase domain
MQTL3B.1	TraesCS3B02G016800	6,956,995–6,958,436	Transferase
MQTL3B.1	TraesCS3B02G017200	7,073,390–7,077,378	Glycosyl transferase, family 14
MQTL3B.1	TraesCS3B02G017800	7,371,289–7,375,600	Pentatricopeptide repeat
MQTL3B.1	TraesCS3B02G018800	8,024,553–8,030,703	Transmembrane protein DDB_G0292058-like
MQTL3B.1	TraesCS3B02G019300	8,133,504–8,137,586	Methyltransferase domain
MQTL3B.1	TraesCS3B02G021900	9,396,614–9,400,783	Glycosyltransferase 61
MQTL3B.1	TraesCS3B02G023700	10,198,656–10,202,090	Glycosyltransferase 61
MQTL3B.1	TraesCS3B02G024400	10,406,614–10,407,459	Gnk2-homologous domain
MQTL3B.1	TraesCS3B02G024500	10,562,122–10,573,523	Protein kinase domain
MQTL3B.1	TraesCS3B02G030500	13,961,374–13,964,727	Protein kinase domain
MQTL3B.1	TraesCS3B02G035200	17,187,124–17,190,917	Cupredoxin
MQTL3B.1	TraesCS3B02G036700	17,974,582–17,975,037	Proteinase inhibitor I12, Bowman-Birk
MQTL3B.1	TraesCS3B02G037000	18,000,322–18,001,131	Proteinase inhibitor I12, Bowman-Birk
MQTL3B.5	TraesCS3B02G158300	153,606,441–153,609,620	Protein kinase domain
MQTL4A.1	TraesCS4A02G025300	17,387,070–17,390,451	Very-long-chain 3-ketoacyl-CoA synthase
MQTL4A.1	TraesCS4A02G026300	18,115,053–18,124,734	Protein kinase domain
MQTL4A.1	TraesCS4A02G026600	18,265,541–18,269,206	Major sperm protein (MSP) domain
MQTL4A.1	TraesCS4A02G026700	18,271,858–18,272,388	Protein kinase domain
MQTL4A.1	TraesCS4A02G029400	21,624,588–21,629,530	P-loop containing nucleoside triphosphate hydrolase
MQTL4A.2	TraesCS4A02G382700	660,790,453–660,795,726	Protein kinase domain
MQTL4A.2	TraesCS4A02G382900	660,916,337–660,921,619	Protein kinase domain
MQTL4B.1	TraesCS4B02G041100	28,414,426–28,418,170	Glycoside hydrolase family 1
MQTL4B.1	TraesCS4B02G042300	28,949,360–28,960,436	Oxysterol-binding protein
MQTL4B.1	TraesCS4B02G042600	29,709,859–29,715,180	AAA + ATPase domain
MQTL4B.1	TraesCS4B02G047100	34,275,331–34,278,683	Protein kinase domain
MQTL4B.7	TraesCS4B02G359300	649,602,163–649,607,404	WD40 repeat
MQTL4D.1	TraesCS4D02G030800	14,514,652–14,515,326	Germin
MQTL4D.1	TraesCS4D02G031600	14,842,844–14,843,518	Germin
MQTL4D.1	TraesCS4D02G031800	14,851,339–14,852,013	Germin
MQTL4D.1	TraesCS4D02G031900	14,857,918–14,858,592	Germin
MQTL4D.1	TraesCS4D02G032000	14,862,810–14,863,484	Germin
MQTL4D.1	TraesCS4D02G032200	14,914,628–14,915,302	Germin
MQTL5A.5	TraesCS5A02G305000	513,959,128–513,961,326	UDP-glucuronosyl/UDP-glucosyltransferase
MQTL5A.5	TraesCS5A02G305100	514,003,067–514,005,001	UDP-glucuronosyl/UDP-glucosyltransferase
MQTL5A.5	TraesCS5A02G306100	514,451,862–514,453,496	S-adenosyl-L-methionine-dependent methyltransferase
MQTL5A.5	TraesCS5A02G306200	515,494,843–515,496,141	Myc-type, basic helix-loop-helix (bHLH) domain
MQTL5B.4	TraesCS5B02G539800	695,480,381–695,486,142	Leucine-rich repeat
MQTL6B	TraesCS6B02G041900	25,911,394–25,916,811	NB-ARC
MQTL6B	TraesCS6B02G051800	30,965,469–30,968,216	Phospholipid/glycerol acyltransferase
MQTL6B	TraesCS6B02G054100	34,219,265–34,220,922	WD40 repeat
MQTL7B.1	TraesCS7B02G013300	9,754,094–9,760,088	Glycosyl transferase, family 31
MQTL7B.2	TraesCS7B02G019600	16,833,162–16,835,197	Rhodanese-like domain
MQTL7D.1	TraesCS7D02G022600	10,593,989–10,595,471	Zinc finger, RING-type
MQTL7D.2	TraesCS7D02G073100	42,835,938–42,838,577	Cytochrome P450

Seedling resistance (SR) and adult plant resistance (APR) are the two basic types of disease resistance employed in breeding programs and examining the expression of CGs in various tissues and developmental stages can help us to identify whether these CGs have a role in SR and/or APR. During the present study, some genes showed significant expression at the seedling stage. Some of these genes (viz., *TraesCS3A02G046000, TraesCS3A02G046100*, and *TraesCS3B02G037000*) encode proteinase inhibitor proteins that are known to confer resistance against several biotic stresses, including fungal pathogens [87]. Another gene, *TraesCS4D02G031800,* encodes a germin-like protein that is supposed to play important roles in the regulation of resistance (by regulating superoxide dismutase activity and the ability to activate the jasmonic acid pathway) to different diseases in plants [85]. The importance of plant secondary metabolite such as glycosyltransferases (e.g., *TraesCS3B02G023700*) in plant–pathogen interactions has also been reported. Glycosyltransferases are known to mediate the hypersensitive responses of plants against fungal infection [88]. Similarly, some genes exhibited significant expression at the reproductive stage, such as *TraesCS1B02G020600*, *TraesCS1B02G020700*, and *TraesCS3B02G024500*. All three genes, viz., *TraesCS1B02G020600*, *TraesCS1B02G020700* and *TraesCS3B02G024500* encode protein kinases that help plants sense various pathogens and activate immunity responses and are also known to be involved in massive transduction pathways upon perception of a pathogen [89]. Using various techniques, including over-expression, gene editing, knockout procedures, or CG-based association mapping, some of the significant CGs discovered in the current study may be validated or functionally characterized.

### MQTL-assisted breeding for stripe rust resistance

The major application of MQTL-assisted breeding is the development of better cultivars with increased disease resistance. Furthermore, major breeding initiatives seek to breed for long-term resistance. MQTLs with reduced CIs and each with multiple QTLs have been recommended for breeding in several earlier studies for different traits such as grain yield and component traits in rice [24], disease resistance in maize [90], anthesis date in wheat [91], and seed quality in soybean [92, 93]. Among the hcMQTLs selected for functional characterization, some MQTLs were also chosen for possible utilization in breeding based on the following criteria: (i) CI < 2 cM, (ii) involvement of at least 5 initial QTLs, (iii) PVE > 12%, and (iv) LOD score > 5. These selected MQTLs (termed as breeder’s MQTLs) included the following:- MQTL1B.1, MQTL2A.1, MQTL2B.4, MQTL2B.5, MQTL3B.3, MQTL3B.5, MQTL4A.1, MQTL4A.2, MQTL5B.3, MQTL6B, MQTL7D.1, and MQTL7D.2. The number of initial QTLs involved in these MQTLs ranged from a minimum of 5 to a maximum of 24 QTLs, PVE values of individual MQTLs ranged from 12.18 to 26.96% and LOD scores ranged from 5.65 to 18.18. Some of these MQTLs included QTLs for both SR and APR. The combination of both the SR and APR has also been found to provide durable resistance against rusts in wheat [94].

### Concluding remarks

In the present study, efforts have been made by us to understand the complex quantitative genetic architecture of stripe rust resistance in wheat by using meta–analysis approach. Our meta-analysis led to the identification of 67 MQTLs, 2 QTL hotspots and 24 singletons associated with stripe rust resistance. More than half of these MQTLs were verified through GWAS-based SNPs/MTAs. It was revealed that many of these MQTLs were found to be co-localized with as many as ten major resistance genes. Among the 67 MQTLs, 29 hcMQTLs were selected and investigated for the detection of CGs. The most promising MQTLs (viz., MQTL1B.1, MQTL2A.1, MQTL2B.4, MQTL2B.5, MQTL3B.3, MQTL3B.5, MQTL4A.1, MQTL4A.2, MQTL5B.3, MQTL6B, MQTL7D.1, and MQTL7D.2) identified in this study may facilitate marker-assisted breeding for stripe rust resistance in wheat. In addition, information on markers flanking the MQTLs can be utilized in genomic selection models to increase the prediction accuracy for stripe rust resistance. Future basic strategic research, including cloning and functional characterization, is suggested for as many as 59 of the 123 DECGs. These CGs can also be utilized for enhancing the wheat resistance against stripe rust after in vivo confirmation/validation using one or more of the following methods: gene cloning, reverse genetic methods, and omics approaches.

## Methods

### QTLs for stripe rust and preparation of input files

An extensive search for publications reporting QTLs associated with stripe rust resistance was performed in wheat using Google Scholar (https://scholar.google.com/) and other available data repositories. This search was further supplemented by a recently developed QTL database [http://wheatqtldb.net/; 23]. The following information was collected from each QTL mapping study: (a) type of mapping population (e.g., F_2:3_, recombinant inbred lines and doubled haploid) and their parents, (b) size of the population, (c) different disease resistance parameters, such as infection rate (IR), disease severity (DS), final disease severity (FDS), area under the disease progress curve (AUDPC), infection type (IT), stripe rust response (SR), normalized difference vegetation index (NDVI), latency period (LP), reaction type (RT), infection response (IR), leaf area infected (LAI), number of stripes per 10 cm^2^ leaf area (SN), (d) pathotype used for phenotyping, (e) method of QTL mapping, (f) position of QTLs and markers flanking the QTLs, (g) logarithm of odds (LOD) scores and (h) *R*^*2*^ values of the individual QTLs. For the meta-analysis, only QTLs with all of the necessary data were selected.

### Construction of the high-density consensus genetic map

For the construction of the consensus map, the R package ‘LPmerge’ was utilized [95]; for this purpose, the following integrated genetic maps were used as the reference maps: (i) the ‘ITMI_SSR map’ with 1406 loci [96] (this map was created by combining 1,184 previously available loci, including 915 RFLPs and 269 SSR markers, with 222 SSR markers developed during the same study); (ii) the ‘Wheat, Consensus SSR map, 2004’ with 1235 marker loci [97] (this map was developed by combining SSR markers from different research groups including the Wheat Microsatellite Consortium, GDM, GWM, CFD, CFA, and BARC); (iii) an integrated map for durum wheat with 30,144 markers [98] (this map included 26,626 SNPs and 791 SSRs); (iv) the 'Illumina iSelect 90 K SNP Array-based genetic map' with 40,267 loci [99] (this map was based on 40,267 SNPs available from the 81,587 markers mapped using wheat 90 K Infinium iSelect SNP array); and (v) the ‘AxiomR, Wheat 660 K SNP array-based genetic map’ with 119,566 markers [100] (this map included 119,001 SNP markers derived from the Wheat660K SNP array, as well as 565 previously available SSR, DArT, STS, SRAP, and ISSR markers; this map has high collinearity with the 90 K and 820 K consensus genetic maps, and it is also consistent with the recently released wheat whole genome assembly). For the construction of the consensus map, markers flanking individual QTLs were also considered. There were hundreds of shared markers present in these genetic maps, albeit at different genetic positions, which were considered when ordering the markers in the consensus chromosomal maps (Supplementary Table 11). The steps involved in the construction of consensus map are well-described in a recent study [101].

### QTL projection on the consensus map

The genetic map file and QTL information file from each study were prepared and utilized as input data text files for QTL projection using BioMercator V4.2 [102]. This software requires a collection of the following different descriptions characterizing each collected QTL: the genetic position of the QTL (peak position and CI), LOD score, *R*^*2*^ value, the trait associated with the QTL and the size of the mapping population utilized for identification of the QTLs. When there was no CI available for a QTL, the CI (95%) was estimated using the population-specific equations provided below [103, 104].

$$\mathrm{For\ the\ }{\mathrm{F}}_{2:3}\mathrm{\ and\ Backcross\ populations},\mathrm{ CI\ }(95\mathrm{\%}) = 530/({\mathrm{R}}^{2}\times \mathrm{ N})$$

$$\mathrm{For\ the\ RIL\ population},\mathrm{ CI\ }(95\mathrm{\%}) = 163/({\mathrm{R}}^{2}\times \mathrm{ N})$$

$$\mathrm{For\ the\ DH\ population},\mathrm{ CI\ }(95\mathrm{\%}) = 287/({\mathrm{R}}^{2}\times \mathrm{ N})$$

According to the statistical approach utilized in the software, the input mapping studies are expected to be independent from each other. Repeated time and place QTL mapping tests usually revealed duplicate QTLs for the same parameter in a few publications. To avoid giving such QTLs too much weight in the meta-analysis, we only kept the QTL with the maximum contribution to the total phenotypic variation. The QTLProj command available in the software permitted the homothetic projection of the peak position and the CIs of the individual QTLs on the consensus map. It is based on a scaling rule between the flanking markers of QTLs on their actual maps and their location on the consensus map [102, 105].

### Prediction of MQTLs through meta-analysis

Meta-analysis of QTLs was performed using the same software BioMercator V4.2 for each individual chromosome, separately. When conducting the analysis, two distinct approaches were used, each of which was determined by the number of QTLs that were available for each chromosome. If there were less than ten QTLs on a single chromosome, the technique described by Goffinet and Gerber [39] was employed; if there were more than ten QTLs on a single chromosome, the second strategy proposed by Veyrieras et al. [38] was used. In the first approach, the model was selected based on their lowest Akaike information criterion (AIC) values. In the second approach, the best model was selected from a list of models that included AIC, AIC3, corrected AIC, Bayesian information criterion (BIC) and average weight of evidence (AWE) models. The model was considered best fit, if it possesses the lowest criteria in at least three of the models.

### Physical mapping of the MQTLs, their validation using GWAS and candidate gene mining

The sequence information of the linked markers flanking the MQTLs was obtained from the publicly available databases like GrainGenes (https://wheat.pw.usda.gov/GG3) or CerealsDB (https://www.cerealsdb.uk.net/cerealgenomics/CerealsDB/indexNEW.php) databases. BLASTN searches against the Wheat Chinese Spring IWGSC RefSeq v1.0 genome assembly in the EnsemblPlants database (http://plants.ensembl.org/index.html) were used to determine the physical positions of markers. The physical positions of some of the SNP markers could also be obtained from the online database “JBrowse-WHEAT URGI database” (https://urgi.versailles.inra.fr/jbrowseiwgsc/). The physical positions of the markers flanking the MQTLs were considered physical/genomic coordinates of the MQTLs.

Furthermore, the data on stripe rust resistance of 20 GWAS published during 2014–2021 were collected and utilized to verify the efficacy of the MQTLs. A summary of these GWAS is provided in Supplementary Table 12. The phenotypic data analyzed in these GWAS were collected from 13 different countries, with population sizes varying from 141 to 23,346, involving different types of wheat, such as durum, spring, winter wheat, and mixed populations of different kinds of wheat. These panels were genotyped with GBS, DArT-seq, and different types of SNP assays, such as 9 K iSelect SNP genotyping array, 20 K Illumina iSelect DNA array, 35 K Axiom array, 90 K Illumina iSelect SNP array, and 660 K SNP array. Similar to ascertaining the physical coordinates of MQTLs, the physical positions of the significant SNPs and/or marker–trait associations (MTAs) identified in these GWAS were obtained either by BLASTN searches, databases or source papers. Given that wheat has a reasonably high linkage disequilibrium (LD) decay distance (about 5 Mb), the MTAs found through GWAS within 5 Mb genomic regions close to a MQTL were accepted as co-located [55].

Furthermore, some high-confidence MQTLs (hcMQTLs) were selected and investigated for the identification of available CGs. These hcMQTLs were chosen based on the following criteria: (i) involvement of at least 3 initial QTLs, (ii) occupying physical distance < 15 Mb, and (iii) genetic distance < 5 cM. The annotated reliable CGs (High Confidence Genes v1.1) within the physical interval of each hcMQTL were retrieved using the BioMart tool of the Ensembl Plants database, and their functional annotations were investigated (https://wheat-urgi.versailles.inra.fr/Seq-Repository/Annotations).

### Expression analysis of the genes available from hcMQTLs

Two expression datasets, NCBI-ID ERP013983 and ERP009837 [36, 106], were used to identify potential genes that were differentially expressed within the hcMQTL intervals based on experiments reported at ExpVIP (http://www.wheat-expression.com) [107]. The ERP009837 dataset consists of differential expression data of 7-day-old seedlings of wheat variety N9134 inoculated with Chinese Pst race CYR 31 with leaf samples collected at 24, 48, and 72 h after inoculation. Leaf samples collected from un-inoculated plants were considered controls.

The ERP013983 dataset contains differential expression data from two genotypes, Vuka (susceptible) and Avocet-Yr5 (resistant), inoculated with Pst strain 87/66 at the three-leaf stage. The leaf samples were taken at 0, 1, 2, 3, 5, 7, 9, and 11 days post-inoculation (dpi) in the susceptible genotype Vuka but only for five days in the resistant line Avocet-Yr5 (at 0, 1, 2, 3, and 5 dpi). The data on gene expression in expVIP were reported as log2 transformed TPM (transcripts per million) values. It is important to mention that only genes with twofold or more up-regulation/down-regulation; calculated by comparing TPM values under stress vs. control) were considered as differentially expressed CGs (DECGs). The results of such DECGs were visualized using the web program Heatmapper, which may be found at http://www.heatmapper.ca/expression/. Furthermore, the transcriptomics data from the Azhurnaya 209-sample RNA sequencing project, which investigated the developmental timeline of a wheat cultivar using a large set of samples from different tissue types [107], were then used in the present study to analyze the expressions of the reported DECGs in different wheat tissues. TPM values were used to assess the level of expressions of CGs within the hcMQTL regions, which was displayed on the heat map using Heatmapper.

### Identification of major *Yr* genes colocalizing with MQTLs

The nucleotide sequences of the genes or sequences of markers linked with* Yr *genes were extracted from the GrainGenes database and BLASTed against the wheat reference genome that is accessible in the EnsemblPlants database. The physical positions of the genes once retrieved were compared with the physical coordinates of MQTLs to ascertain their co-localization with the MQTLs.

## Supplementary Information

**Additional file 1: Supplementary Table 1.** QTLs utilized in the present study for meta-analysis. **Supplementary Table 2.** Detailed information on MQTLs identified during the present study. **Supplementary Table 3.** Singletons detected during the present study. **Supplementary Table 4.** QTL hotspots detected during the present study. **Supplementary Table 5.** Validation of MQTLs with significant SNPs or MTAs identified in earlier GWAS. **Supplementary Table 6.** MQTLs co-localizing with known major rust resistance genes. **Supplementary Table 7.** Gene models available from hcMQTL regions. **Supplementary Table 8.** Differentially expressed candidate genes identified within the hcMQTL regions. **Supplementary Table 9.** Comparison of our study with the study previously published by Jan et al. [54]. **Supplementary Table 10.** Details on recently identified QTLs and their association with the MQTLs identified during the present study. **Supplementary Table 11.** Markers available from different genetic maps utilized for the construction of consensus map (shared markers are highlighted with dark red colour). **Supplementary Table 12.** Summary of genome-wide association studies considered in the current study.

**Additional file 2: Supplementary Fig. 1.** Expression of selected most promising candidate genes in different wheat tissues.

## Data Availability

All data generated or analysed during this study are included in this published article [and its additional files]. The Supplementary Information given below.

## Abbreviations

QTL: Quantitative trait loci
M-QTL: Meta- Quantitative trait loci
Yr: Yellow rust
GWAS: Genome wide association study
