# Supplementary material for: Comprehensive meta-QTL analysis for dissecting the genetic architecture of stripe rust resistance in bread wheat
Source: BMC Genomics. 2023 May 12;24:259. doi: 10.1186/s12864-023-09336-y (PMC10182688; doi:10.1186/s12864-023-09336-y)
Supplement: Supplementary file 2 — Additional file 2: Supplementary Fig. 1. Expression of selected most promising candidate genes in different wheat tissues. [file 12864_2023_9336_MOESM2_ESM.pdf]

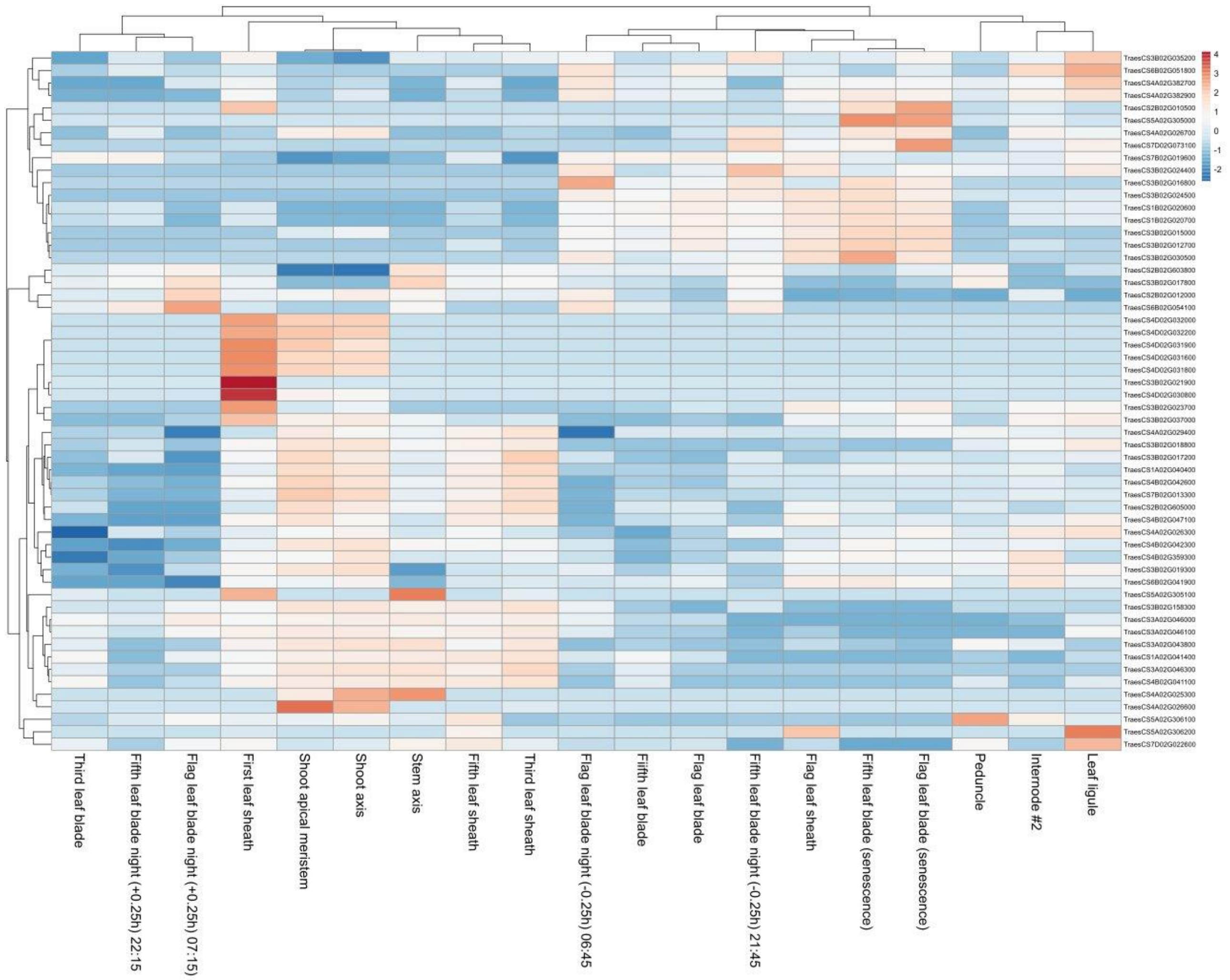

Supplementary Fig. 1 Expression of selected most promising candidate genes in different wheat tissues
